# Transcriptome analysis of three medicinal plants of the genus *Polygonatum*: identification of genes involved in polysaccharide and steroidal saponins biosynthesis

**DOI:** 10.3389/fpls.2023.1293411

**Published:** 2023-11-17

**Authors:** Jimei Lu, Jinchen Yao, Jingzhe Pu, Duomei Wang, Junling Liu, Yazhong Zhang, Liangping Zha

**Affiliations:** ^1^ College of Pharmacy, Anhui University of Chinese Medicine, Hefei, China; ^2^ Anhui Institute for Food and Drug Control, Hefei, China

**Keywords:** Polygonati Rhizoma, transcriptome, polysaccharides biosynthesis, steroidal saponins biosynthesis, transcription factor

## Abstract

Polysaccharides and saponins are the main active components of Polygonati Rhizoma. Studying the molecular mechanism of their synthesis pathway is helpful in improving the content of active components at the molecular level. At present, transcriptome analysis of three *Polygonatum* species (*Polygonatum sibiricum* Red*., Polygonatum cyrtonema* Hua, *Polygonatum kingianum* Coll. et Hemsl.) has been reported, but no comparative study has been found on the transcriptome data of the three species. Transcriptome sequencing was performed on the rhizomes of three *Polygonatum* species based on high-throughput sequencing technology, and all transcripts were assembled. A total of 168,108 unigenes were generated after the removal of redundancy, of which 121,642 were annotated in seven databases. Through differential analysis and expression analysis of key enzyme genes in the synthesis pathway of three *Polygonatum* polysaccharides and steroidal saponins, 135 differentially expressed genes encoding 18 enzymes and 128 differentially expressed genes encoding 28 enzymes were identified, respectively. Numerous transcription factors are involved in the carbohydrate synthesis pathway. Quantitative real-time PCR was used to further verify the gene expression level. In this paper, we present a public transcriptome dataset of three medicinal plants of the genus *Polygonatum*, and analyze the key enzyme genes of polysaccharide and steroidal saponins synthesis pathway, which lays a foundation for improving the active component content of Polygonati Rhizoma by molecular means.

## Introduction

Polygonati Rhizoma is a traditional Chinese herb used in both food and medicine, and has a medicinal history of more than 2000 years (Hu et al., 2022; Li et al., 2023). In China, there are about 39 species of the genus *Polygonatum*. Among them, *Polygonatum cyrtonema* Hua (*P. cyrtonema*), *Polygonatum kingianum* Coll. et Hemsl. (*P. kingianum*) and *Polygonatum sibiricum* Red. (*P. sibiricum*) were included in Chinese Pharmacopoeia (2020 edition) as the original plants of Polygonati Rhizoma. Modern pharmacological studies have shown that polysaccharide and saponin, the main active components of Polygonati Rhizoma, have a series of important functions such as anti-aging, immune regulation, anti-tumor, hypoglycemic (Long et al., 2018; Zhao et al., 2018; Shen et al., 2021; Shen et al., 2022).

*Polygonatum* polysaccharide is a class of biological macromolecules composed of a variety of monosaccharides in different proportions. The researchers revealed that there are differences in polysaccharide structure, monosaccharide type, and content in different types and origins of Polygonati Rhizoma, which means that its biological activities also have certain differences (Wang et al., 2022). Comparative studies of polysaccharides from *P. cyrtonema* (PCP), *P. kingianum* (PKP), and *P. sibiricum* (PSP) showed that their physicochemical properties, antioxidant activity *in vitro*, and the regulation of macrophage polarization were different (Bai et al., 2021). Previous studies have shown that UDP-glucose is the basis for the production of multiple NDP-monosaccharides in the biosynthesis pathway of polysaccharides, and is also the main form of activated sugar in higher plants. It is directly generated by sucrose under the action of sucrose synthase (SUS) or is catalyzed by UDP-glucose pyrophosphorylase (UGPase) to produce using glucose 1-phosphate as the substrate (Decker et al., 2017; Wang et al., 2017). UDP-glucose generates a series of NDP-sugar under the action of UDP-glucose 4-epimerase (GALE), UDP-glucose dehydrogenase (UGDH), and other enzymes. At the same time, sucrose is catalyzed by enzymes such as hexokinase (HK), fructokinase (scrK), mannose-6-phosphate isomerase (MPI), and phosphomannose isomerase (PMM) to generate GDP-mannose and GDP-fucose. These monosaccharides are finally incorporated into polysaccharide polymers by glycosyl transferases (GTs) to form characteristic polysaccharides. At present, research on the synthesis pathway of polysaccharides has been reported in medicinal plants such as *Panax ginseng* (Fang et al., 2022), *Dendrobium moniliform* (Yuan et al., 2019), and *Codonopsis pilosula* (Gao et al., 2015).

Saponins include steroid saponins and triterpenoid saponins, which are composed of one or more hydrophilic sugar residues and hydrophobic sapogenin. Steroidal saponins are mainly present in plants such as the Liliaceae (Luo et al., 2018), Smilaxaceae (Tian et al., 2017), Asparagaceae, etc.(Sidana et al., 2016), and their anti-tumor activity has been widely studied (Mimaki et al., 1994; Lu et al., 2009; Han et al., 2018). The biosynthesis process of steroidal saponins is divided into three stages, and their upstream synthesis includes the cytoplasmic mevalerate (MVA) pathway and the plastid methyl-ethrolitol 4-phosphate (MEP) pathway, which eventually produces mutually transferable isopentenyl pyrophosphate (IPP) and dimethylallyl diphosphate (DMAPP). In the action of geranyl diphosphate synthase (GPS), farnesyl diphosphate synthase (FPS), squalene synthase (SQS), and squalene epoxidase (SE), IPP and DMAPP generated 2,3-oxidosqualene. Cycloartenol synthase (CAS) catalyzes 2,3-oxidosqualene to cycloartenol, the precursor of steroidal sapogenin, which undergoes a series of reactions including oxidation, hydroxylation, and glycosylation to ultimately generate steroidal saponins (Upadhyay et al., 2018; Guo et al., 2021). Some studies have shown that lanosterol can participate in the synthesis of plant sterols and steroid glycoalkaloids. However, the current theory is more accepted that cycloartenol is converted to sitosterol, and then steroidal saponins are produced by hydroxylase and glycosyltransferase (Chen et al., 2021). At present, the genes related to plant steroidal saponins biosynthesis need to be further studied. It has been reported that the expression levels of key regulatory and rate-limiting enzyme 3-hydroxy-3-methylglutaryl-coenzyme A reductase (HMGR) in the MVA pathway can affect the content of monoglycosylated saponins in *Medicago truncatula* (Moses et al., 2014). Therefore, identifying the key genes in the synthesis pathway of steroidal saponins is of great significance for understanding the synthesis mechanism of steroidal saponins.

Transcriptomics takes all transcripts in the sample as the research object. It plays an important role in revealing the molecular mechanism of biological processes and mining functional genes. It is the most widely used and rapidly developing omics technology (Lowe et al., 2017). Since RNA sequencing technology was proposed, it has gradually become the first choice for gene expression analysis due to its advantages such as high sensitivity, high accuracy, high repeatability, and not limited by the completeness of genome data of species (Su et al., 2014; Imadi et al., 2015). It has been widely used in transcriptome analysis of species such as *Panax japonicus* (Rai et al., 2016), *Carthamus tinctorius* L. (Huang et al., 2012), and *Atractylodes lancea* (Ahmed et al., 2016). Based on the transcriptome data mining of *P. cyrtonema*, *P. kingianum*, and *P. sibiricum*, scholars have obtained multiple key enzyme genes related to the synthesis of polysaccharides, saponins, and flavonoids, which is conducive to improving the content of effective components of polygonati rhizoma through genetic engineering (Wang et al., 2019; Yang et al., 2019; Han et al., 2023). However, at present, comparative studies on the biological activities and synthesis pathways of three *Polygonatum* polysaccharides and steroidal saponins are relatively lacking. In this paper, through sequencing and analysis of the transcriptome of *P. cyrtonema*, *P. kingianum*, and *P. sibiricum*, the key enzyme genes affecting the content differences of *Polygonatum* polysaccharides and steroidal saponins were explored, laying a foundation for the regulation of the genes of *Polygonatum* polysaccharide and steroidal saponins synthesis pathway.

## Materials and methods

### Plant materials and total RNA extraction

*P. cyrtonema* and *P. sibiricum* were collected from Guoziyuan Township, Jinzhai County, Lu’an City, Anhui Province, and *P. kingianum* was collected from Zhangshan Village, Xinghua Township, Hong’an County, Huanggang City, Hubei Province. Three plants of each variety were taken as replicates. The test materials were identified by associate researcher Liangping Zha (Anhui University of Chinese Medicine) as Liliaceae family plants *P. cyrtonema*, *P. sibiricum*, and *P. kingianum.* The rhizomes of the plants were collected, cleaned with sterilized water, dried on filter paper, snap-frozen in liquid nitrogen, and stored at -80°C. According to the manufacturer’s instructions, the sample was first ground into a powder with liquid nitrogen, and total RNA was extracted using CTAB-PBIOZOL and the ethanol precipitation protocol. The RNA concentration, 28S/18S, and RNA integrity number (RIN) were detected using the Standard Sensitivity RNA Analysis Kit, which was performed by the Fragment Analyzer.

### Extraction and determination of total polysaccharide and saponins

Rhizomes of three *Polygonatum* plants were dried in an oven at 60°C to constant weight, ground into powder, and passed through an 80 mesh sieve. The extraction method of polysaccharides was as described in Chinese Pharmacopoeia. The absorbance was measured at 574 nm, D-glucose was used as a reference, and total polysaccharide was determined using the anthrone-sulfuric acid method. The extraction method of *Polygonatum* saponin refers to the method of Hu et al. and saponin content was analyzed using the 5% vanillin-glacial acetic acid–perchloric acid method, diosgenin as the reference, the wavelength was measured at 530 nm (Hu et al., 2022).

### RNA−Seq library construction and sequencing

The mRNA with polyA of the three varieties of polygonati rhizoma (three replicates) was enriched using Oligo (dT) magnetic beads, cleaved into short fragments, and cDNA strands were obtained by reverse transcription with random N6 primers. The double-stranded cDNA ends were repaired, the 5’ end was phosphorylated and a single nucleotide A was added at the 3’ end. Then, cDNA was ligated to the sequencing adapters, and the ligated products were amplified by PCR to construct a cDNA library and sequenced on the DNBSEQ platform (Beijing Genomics Institute, Shenzhen, China).

The SOAPnuke filtering software removed reads containing adapters, unknown nucleotides content greater than 5%, and low-quality (more than 20% of bases with quality <10). *De novo* assembly and redundant sequence removal were performed in Trinity and Tgicl, respectively.

### Functional annotation

To obtain more comprehensive information on gene function, the assembled unigenes were annotated to seven databases, including the NCBI non-redundant nucleotide sequence (NT, ftp://ftp.ncbi.nlm.nih.gov/blast/db), NCBI non-redundant protein sequences (NR, ftp://ftp.ncbi.nlm.nih.gov/blast/db), Kyoto Encyclopedia of Genes and Genomes (KEGG, http://www.genome.jp/kegg), Clusters of euKary-otic Orthologous Groups (KOG, http://www.ncbi.nlm.nih.gov/KOG), SwissProt (http://ftp.ebi.ac.uk/pub/databases/swissprot), Gene Ontology (GO, http://geneontology.org) and Pfam (http://pfam.xfam.org). Blast2GO software (version 2.5.0) was used to further perform GO annotation and functional analysis of unigenes annotated to the NR database, and the annotation results were divided into three parts: molecular function, cellular component, and biological process.

### Identification of differentially expressed genes

Clean reads were matched to reference gene sequences by Bowtie2 software (version 2.2.5), and then gene expression levels were calculated for each sample using RSEM software (version 1.2.8). Transcripts per kilobase per million fragments (FPKM) were used to normalize read counts of transcripts. DEGseq2 (version 1.4.5) software was applied to the analysis of DEGs. Genes with Q value ≤ 0.001 and |log_2_FC|>1 were defined as DEGs. DEGs enrichment in GO terms and KEGG pathways were determined using the phyper function with Q value ≤0.05.

### Analysis of genes encoding transcription factors

TFs can regulate the transcription level of genes by binding to promoters. In order to analyze the TF family of the transcriptome of three *Polygonatum* plants, getorf software (EMBOSS:6.5.7.0) was used to detect the open reading frame (ORF) of Unigene, and hmmsearch (version 3.0) searched for proteins with certain gene family domains in candidate sequences by comparing ORFs. Then the genes encoding TFs were identified based on PlantTFDB.

### Bioinformatics analysis of UDP-apiose/xylose synthase genes

DNAMAN software was used for homologous amino acid alignment, and SWISS MODEL (http://swissmodel.expasy.org/) and PyMOL software to predict the tertiary structure of the proteins. The phylogenetic tree was constructed in MEGA7.0 software using the neighbor-joining method.

### Quantitative real−time PCR validation

Total RNA from the three *Polygonatum* plants was reverse transcribed using the All-in-One First-Strand Synthesis MasterMix kit (LABLEAD, China). The qRT-PCR was performed using the Bio-Rad CFX96 platform (Bio-Rad, USA) according to the instructions of the 2×Realab Green PCR Fast mixture kit. The mRNA abundance of the target genes was normalized to the mRNA abundance of the internal reference gene *EF-1α2* (Yang et al., 2020), and the relative gene expression analysis was performed using the 2^-ΔΔCt^ method. The specific primers were designed using the Primer software (version 5.0).

## Results

### Total polysaccharide content of *Polygonatum* samples

**Figure 1:**
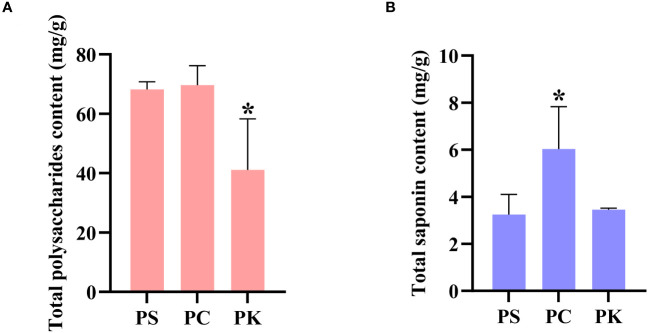
Contents of polysaccharides and saponins in *P. sibiricum, P. cyrtonema*, and *P. kingianum* (Mean values ± SD, n = 3). **(A)** Contents of polysaccharides. **(B)** Contents of saponins. *data compared with the *P. sibiricum*, P < 0.05. PS, PC, and PK represent *P. sibiricum, P. cyrtonema*, and *P. kingianum*, respectively.

We used the anthrone-sulfuric acid method to determine the polysaccharide content of three *polygonatum* species, with three replicates per sample, expressed as the mean value with standard deviation. The results showed that the polysaccharide content of *P. sibiricum* and *P. cyrtonema* were almost the same, both significantly higher than that of *P. kingianum*, 1.66 and 1.70 times that of *P. kingianum*, respectively (**Figure 1A**).

### Total saponin content of *Polygonatum* samples

As the main active secondary metabolite of polygonati rhizoma, total saponin has attracted much attention. The vanillin-glacial acetic acid method was used to determine the total saponin content of three botanical origins of polygonati rhizoma. The results showed that the total saponin content of *P. cyrtonema* was significantly higher than that of *P. sibiricum* and *P. kingianum*, which were 1.86 and 1.75 times higher, respectively. The total saponin content of *P. kingianum* was slightly higher than that of *P. sibiricum*, which was 1.07 times that of *P. sibiricum* (**Figure 1B**).

### Transcriptome analysis based on RNA−Seq

Nine samples of *P. sibiricum*, *P. cyrtonema*, and *P. kingianum* (three replicates for each species) were sequenced on the DNBSEQ platform, and the raw reads were filtered by the filtering software SOAPnuke to obtain clean reads of 128.07 M, 127.72 M, and 128.11 M respectively. Each sample yielded more than 6.32 Gb of clean data. The Q30 percentages were more than 92.52%. Using Tgicl to cluster the transcripts to remove redundancy, a total of 168,108 unigenes were obtained, a mean length of 1,236 base pairs (bp) and a mean N50 of 1,839 bp, and the average GC content was 43.61%. Among them, 70.57% (118,640) and 46.95% (78,927) unigenes were longer than 500 bp and 1000 bp, respectively (**Table S1**; **Figure S1**).

### Functional annotation and expression overview of unigenes

**Figure 2:**
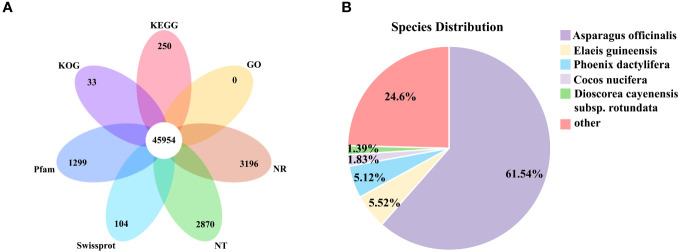
Transcriptome annotation. **(A)** Venn diagram of functional annotation of seven public protein databases (NR, NT, SwissProt, Pfam, KOG, KEGG and GO). **(B)** Species distribution of homologous sequences against Nr database.

The assembled unigenes were compared with NR, NT, Swissprot, KEGG, KOG, Pfam, and GO databases. 27.34% of the unigenes were mapped simultaneously in the seven databases, and 72.36% of the unigenes were mapped to at least one public database (**Table 1**). Among the six databases of NR, NT, SwissProt, Pfam, KOG, and KEGG, there were 3196, 2870, 104, 1299, 33, and 250 unique unigenes, respectively, and unique genes were not annotated in the GO database (**Figure 2A**).

**Table 1:** Functional annotation results from seven public databases.

Databases	Total	NR	NT	SwissProt	KEGG	KOG	Pfam	GO	Intersection	Overall
Number	168,108	116,279	91,919	88,252	91,738	92,091	85,079	86,872	45,954	121,642
Percentage(%)	100	69.17	54.68	52.50	54.57	54.78	50.61	51.68	27.34	72.36

The 116,279 unigenes annotated in NR database were subjected to homology analysis, among which the highest homology was *Asparagus officinalis* (61.54%). Followed by *Elaeis guineensis* (5.52%), *Phoenix dactylifera* (5.12%), *Cocos nucifera* (1.83%), *Dioscorea cayenensis subsp. Rotundata* (1.39%) (**Figure 2B**).

**Figure 3:**
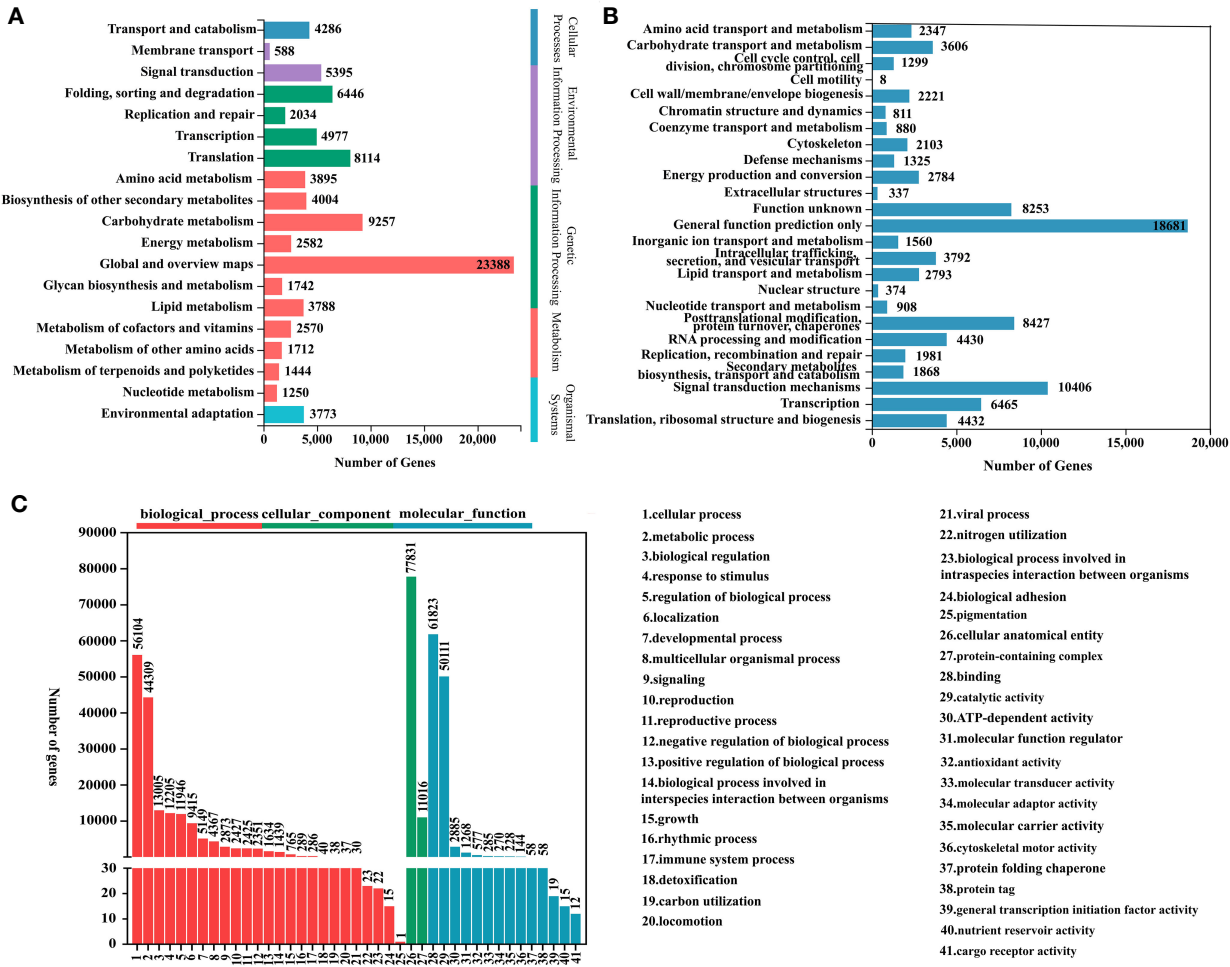
Functional annotations for three *Polygonatum* plants. **(A)** KEGG pathway classification. **(B)** KOG functional classification. **(C)** GO functional classification.

In order to further explore the interactions and biological functions of genes in polygonati rhizoma, a total of 91,738 unigenes were mapped to the KEGG database and assigned to five categories: genetic information processing, environmental information processing, cellular processes, metabolism, and organismal systems. It contains 19 subcategories such as “carbohydrate metabolism”, “biosynthesis of other secondary metabolites”, “transport and catabolism”, among which 11 subcategories belong to the “metabolism” category (**Figure 3A**; **Table S2**). The results of KEGG enrichment showed that the “carbohydrate metabolism” subcategory involved 15 pathways. The “pentose and glucuronate interconversions” pathway contains the largest number of unigenes (2,179). Eighteen pathways were assigned to the “biosynthesis of other secondary metabolites” subcategory, and “phenylpropanoid biosynthesis” was the most abundant pathway containing unigenes. Nine pathways were involved in “metabolism of terpenoids and polyketides” subcategory, most unigenes (426) were enriched in “terpenoid backbone biosynthesis”, 211 unigenes were enriched in “Sesquiterpenoid and triterpenoid biosynthesis” pathway (**Figure S2**).

Based on the NR database annotation results, the 86,872 transcripts mapped to the GO database were classified into three categories: biological process, molecular function, and cellular component by the Blast2GO software. Among them, biological process was the largest functional category, and the main terms were “cellular process” (GO:0009987; 56,104 genes) and “metabolic process” (GO:0008152; 44,309 genes). In the cell component and molecular function categories, “cellular anatomical entity” (GO:0110165; 77,831 genes) and “binding” (GO:0005488; 61,823 genes) were the richest terms, respectively (**Figure 3C**).

The KOG database alignment showed that 92,091 unigenes were divided into 25 functional clusters, and that the “general function prediction only” had the largest number of unigenes (18,681), followed by “signal transduction mechanisms”, including 10,406 unigenes (**Figure 3B**).

Unigenes with FPKM>1 were analyzed. The results showed that 48,397, 55,259 and 53,099 unigenes were expressed in *P. sibiricum*, *P. cyrtonema*, and *P. kingianum*, respectively (**Figure S3A**). The boxplot showed that the expression level of *P. kingianum* was slightly higher than that of *P. sibiricum* and *P. cyrtonema* (**Figure S3B**).

### Identification of differentially expressed genes

**Figure 4:**
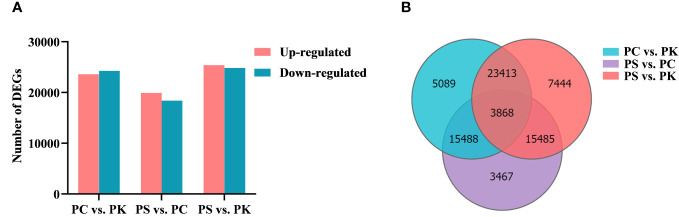
Analysis of DEGs. **(A)** Upregulated and downregulated DEGs in different samples. **(B)** Venn diagram of DEGs in diverse comparison groups.

The screening of DEGs is helpful in analyzing the important functional genes of polygonati rhizoma. Taking Q ≤ 0.001 as the screening condition of DEGs, 47,858 DEGs were selected from the *P. cyrtonema* vs *P. kingianum* analysis, including 23,596 up-regulated genes and 24,262 down-regulated genes. 38,308 DEGs were detected by comparing the expression levels of *P. sibiricum* and *P. cyrtonema*, with 19,915 up-regulated genes and 18,393 down-regulated genes. The comparison of expression levels between *P. sibiricum* and *P. kingianum* identified 50,210 DEGs, including 25,380 up-regulated genes and 24,830 down-regulated genes (**Figure 4A**). The analysis of DEGs showed that there were 7444 specific DEGs in *P. sibiricum* vs *P. kingianum*, and then there were 5089 and 3467 specific DEGs in *P. cyrtonema* vs *P. kingianum* and *P. sibiricum vs P. cyrtonema*, respectively. In addition, a total of 3868 DEGs were detected among the three comparison groups (**Figure 4B**).

In order to further analyze the DEGs, KEGG and GO enrichment were performed using phyper function in R software. In KEGG analysis, *P. cyrtonema* vs *P. kingianum, P. sibiricum* vs *P. kingianum, P. sibiricum* vs *P. cyrtonema* identified 20,258, 21,180, and 16,154 DEGs, respectively. In the *P. cyrtonema* vs *P. kingianum*, there were 220, 368, 757 and 677 DEGs enriched to fructose and mannose metabolism (ko00051) pathway, galactose metabolism (ko00052) pathway, starch and sucrose metabolism (ko00500) pathway, amino sugar and nucleotide sugar metabolism (ko00520) pathway related to polysaccharide biosynthesis, respectively. Among them, the fructose and mannose metabolism (ko00051) pathway and galactose metabolism (ko00052) pathway were included in the top 20 pathways. In *P. sibiricum* vs *P. kingianum*, 256, 378, 828, and 691 DEGs were enriched to ko00051, ko00052, ko00500, and ko00520 pathways, respectively, and only fructose and mannose metabolism (ko00051) pathway was included in the top 20 pathways. 174, 278, 601, and 526 DEGs in *P. sibiricum* vs *P. cyrtonema* were enriched in the above four polysaccharide synthesis pathways, respectively. In conclusion, the largest number of DEGs enriched to starch and sucrose metabolism (ko00500) pathway, however, the higher enrichment of fructose and mannose metabolism (ko00051) pathway in both *P. cyrtonema* vs *P. kingianum* and *P. sibiricum* vs *P. kingianum*, this pathway can be focused on in subsequent studies of polysaccharide metabolism. Among the three pathways of terpenoid backbone biosynthesis (ko00900), sesquiterpenoid and triterpenoid biosynthesis (ko00909) and steroid biosynthesis (ko00100) associated with the synthesis of steroidal saponins, the largest number of DEGs were enriched to sesquiterpenoid and triterpenoid biosynthesis (ko00909) pathway. The most enriched pathway among the three comparison groups was “ribosome”. In GO enrichment analysis, the most expansive GO term among the different comparison groups was “RNA binding” (**Figure S4**).

### Putative DEGs involved in polysaccharide biosynthesis

**Figure 5:**
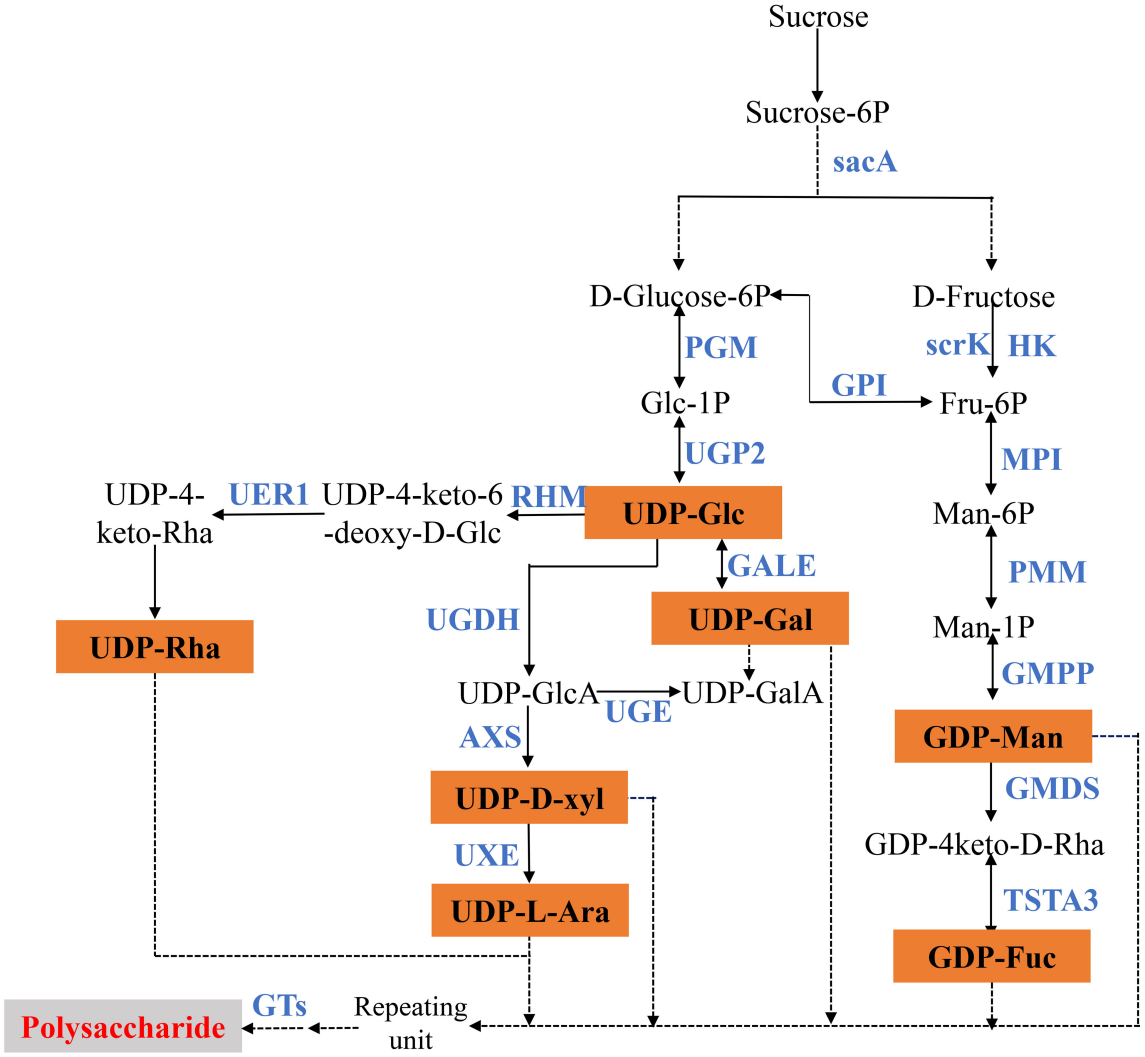
Proposed pathways for polysaccharide biosynthesis in three *Polygonatum* plants.

**Figure 6:**
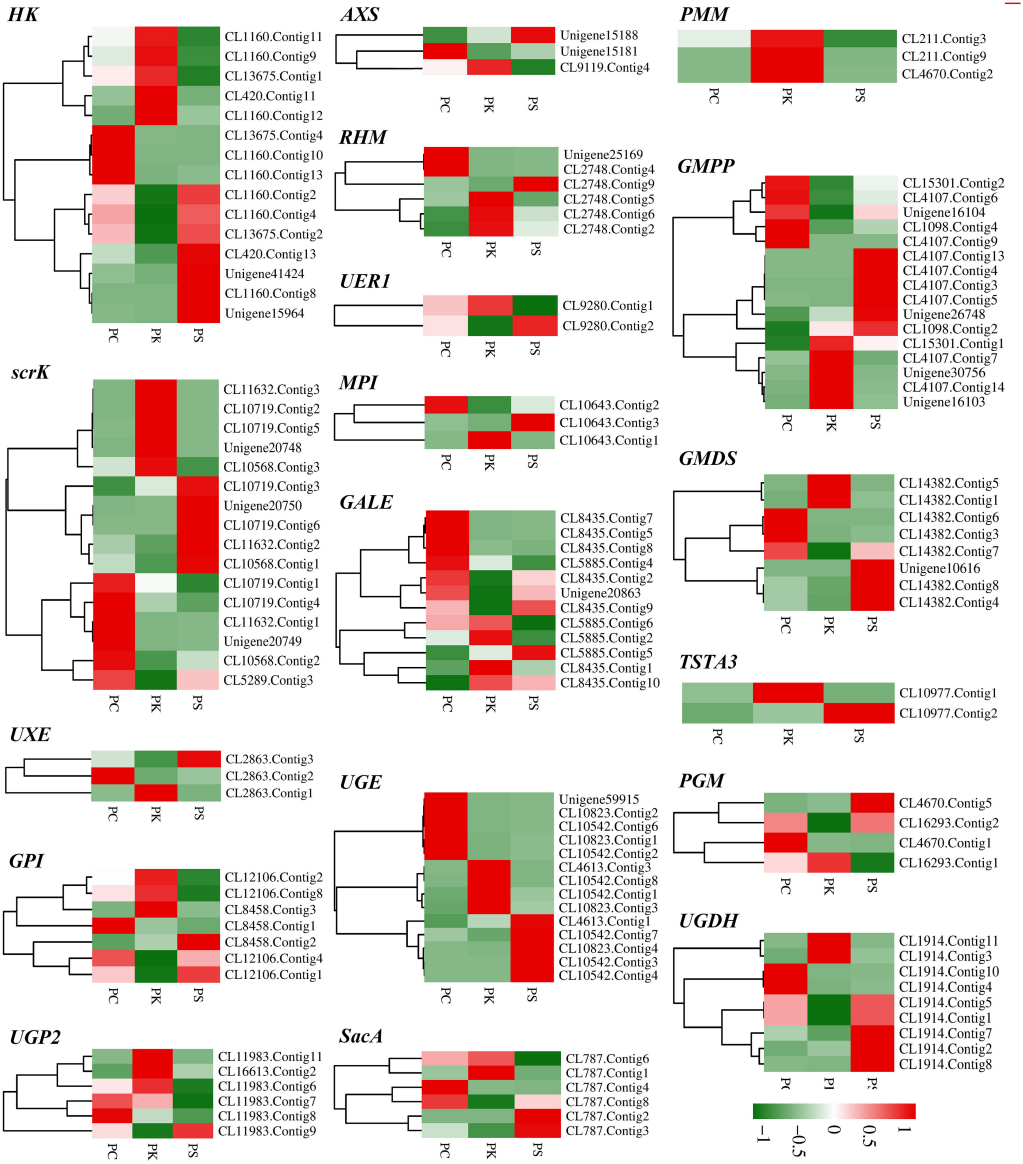
Relative expression patterns of unigenes encoding enzymes involved in polysaccharide biosynthesis.

Polysaccharide is the main active component of polygonati rhizoma. Based on the KEGG enrichment results, we identified the genes encoding key enzymes on the starch and sucrose metabolism and amino sugar and nucleotide sugar metabolism pathways. After removing the sequences with FPKM<1, incomplete sequences, and completely consistent sequences, we ultimately obtained 163 genes encoding 18 key enzymes for *polygonatum* polysaccharide biosynthesis, of which 135 were further identified as DEGs, including mannose-1-phosphate guanylyltransferase (GMPP, 16 unigenes), scrK (16 unigenes), UDP-glucuronate 4-epimerase (UGE, 14 unigenes), HK (15 unigenes) et al. (**Table 2**; **Figure 5**). Heat maps were drawn based on FPKM values to reflect the expression levels of these genes in *P. cyrtonema*, *P. kingianum* and *P. sibiricum* (**Figure 6**). The expression levels of 28 genes encoding key enzymes were higher in *P. sibiricum* and *P. cyrtonema*, while the expression levels were the lowest in *P. kingianum*. Among them, the expression levels of 13 genes encoding 8 key enzymes-AXS (Unigene15181), GMPP (CL15301.Contig2, CL4107.Contig6, Unigene16104, CL1098.Contig4), MPI (CL10643.Contig2), scrK (CL10568.Contig2, CL5289.Contig3), GALE (CL8435.Contig2, Unigene20863), GMDS(CL14382.Contig7), GPI (CL12106.Contig4) and SacA (CL787.Contig8) were the highest in *P. cyrtonema*, suggesting that they play an important role in the polysaccharide synthesis pathway of polygonati rhizoma.

**Table 2:** Number of unigenes encoding key enzymes involved in polysaccharide biosynthesis in three *Polygonatum* plants.

Enzyme name	EC number	Unigene Number	DEGs Number
beta-fructofuranosidase (sacA)	3.2.1.26	9	6
Hexokinase (HK)	2.7.1.1	17	15
Fructokinase (scrK)	2.7.1.4	19	16
mannose-6-phosphate isomerase (MPI)	5.3.1.8	3	3
Phosphomannomutase (PMM)	5.4.2.8	3	3
mannose-1-phosphate guanylyltransferase (GMPP)	2.7.7.13	20	16
GDP-mannose 4,6-dehydratase (GMDS)	4.2.1.47	8	8
GDP-L-fucose synthase (TSTA3)	1.1.1.271	3	2
glucose-6-phosphate isomerase (GPI)	5.3.1.9	8	7
Phosphoglucomutase (pgm)	5.4.2.2	5	4
UTP-glucose-1-phosphate uridylyltransferase (UGP2)	2.7.7.9	7	6
UDP-glucose 4-epimerase (GALE)	5.1.3.2	12	12
UDP-glucuronate 4-epimerase (UGE)	5.1.3.6	18	14
UDP glucose 6-dehydrogenase (UGDH)	1.1.1.22	11	9
UDP-apiose/xylose synthase (AXS)	AXS	5	3
UDP-arabinose 4-epimerase (UXE)	5.1.3.5	5	3
UDP-glucose 4,6-dehydratase (RHM)	4.2.1.76	8	6
3,5-epimerase/4-reductase (UER1)	5.1.3-,1.1.1-	2	2

### Putative DEGs involved in steroidal saponins biosynthesis

The upstream pathway of steroidal saponins synthesis is the same as that of terpenoid skeleton synthesis, including MVA and MEP pathways. The downstream pathway includes the biosynthesis of steroid sapogenin precursor sterols and the oxidation and modification of steroidal saponins. Terpenoid backbone biosynthesis (ko00900), sesquiterpenoid and triterpenoid biosynthesis (ko00909), steroid biosynthesis (ko00100) were the three metabolic pathways of steroidal saponins synthesis. After screening FPKM>1 and complete sequences, 155 unigenes encoding key enzymes of steroidal saponins were obtained, of which 128 were further identified as DEGs (**Table 3**).

**Table 3:** Number of unigenes encoding key enzymes involved in steroidal saponins biosynthesis in three *Polygonatum* plants.

Enzyme name	EC number	unigene number	DEGs Number
Acetyl-CoA C-acetyltransferase (AACT)	2.3.1.9	7	5
Hydroxymethylglutaryl CoA synthase (HMGS)	2.3.3.10	6	6
3-hydroxy-3-methylglutaryl-coenzyme A reductase (HMGR)	1.1.1.34	10	9
Mevalonate kinase (MVK)	2.7.1.36	5	5
Phosphomevalonate kinase (PMVK)	2.7.4.2	4	3
Diphosphomevalonate decarboxylase (MVD)	4.1.1.33	4	2
1-deoxy-D-xylulose-5-phosphate synthase (DXS)	2.2.1.7	11	7
1-deoxy-D-xylulose-5-phosphate reductoisomerase (DXR)	1.1.1.267	2	2
2-C-methyl-d-erythritol 4-phosphate cytidylyltransferase (MCT)	2.7.7.60	2	2
4-diphosphocytidyl-2-C-methyl-D-erythritol kinase (CMK)	2.7.1.148	3	2
2-C-methyl-D-erythritol 2,4-cyclodiphosphate synthase (MCS)	4.6.1.12	2	2
(E)-4-hydroxy-3-methylbut-2-enyl-diphosphate synthase (HDS)	1.17.7.1	3	3
4-hydroxy-3-methylbut-2-enyl diphosphate reductase (HDR)	1.17.7.4	3	3
Isopentenyl-diphosphate Delta-isomerase (IDI)	5.3.3.2	10	8
Farnesyl diphosphate synthase (FPPS)	2.5.1.1,2.5.1.10	3	2
Squalene synthase (SS)	2.5.1.21	9	8
Squalene epoxidase (SE)	1.14.14.17	6	5
Cycloartenol synthase (CAS)	5.4.99.8	2	2
Sterol 24-C-methyltransferase (SMT1)	2.1.1.41	3	2
Plant 4,4-dimethylsterol C-4 alpha-methyl-monooxygenase (SMO1)	1.14.18.10	9	5
Cycloeucalenol cycloisomerase (CPI1)	5.5.1.9	8	8
Delta 14-sterol reductase (FK)	1.3.1.70	5	5
Cholestenol Delta-isomerase (HDY1)	5.3.3.5	6	6
24-methylenesterol C-methyltransferase (SMT2)	2.1.1.143	10	10
Plant 4 alpha-monomethylsterol monooxygenase (SMO2)	1.14.18.11	5	4
Delta 7-sterol 5-desaturase (STE1)	1.14.19.20	8	6
7-dehydrocholesterol reductase (DWF5)	1.3.1.21	5	4
Delta 24-sterol reductase (DWF1)	1.3.1.72	4	2

**Figure 7:**
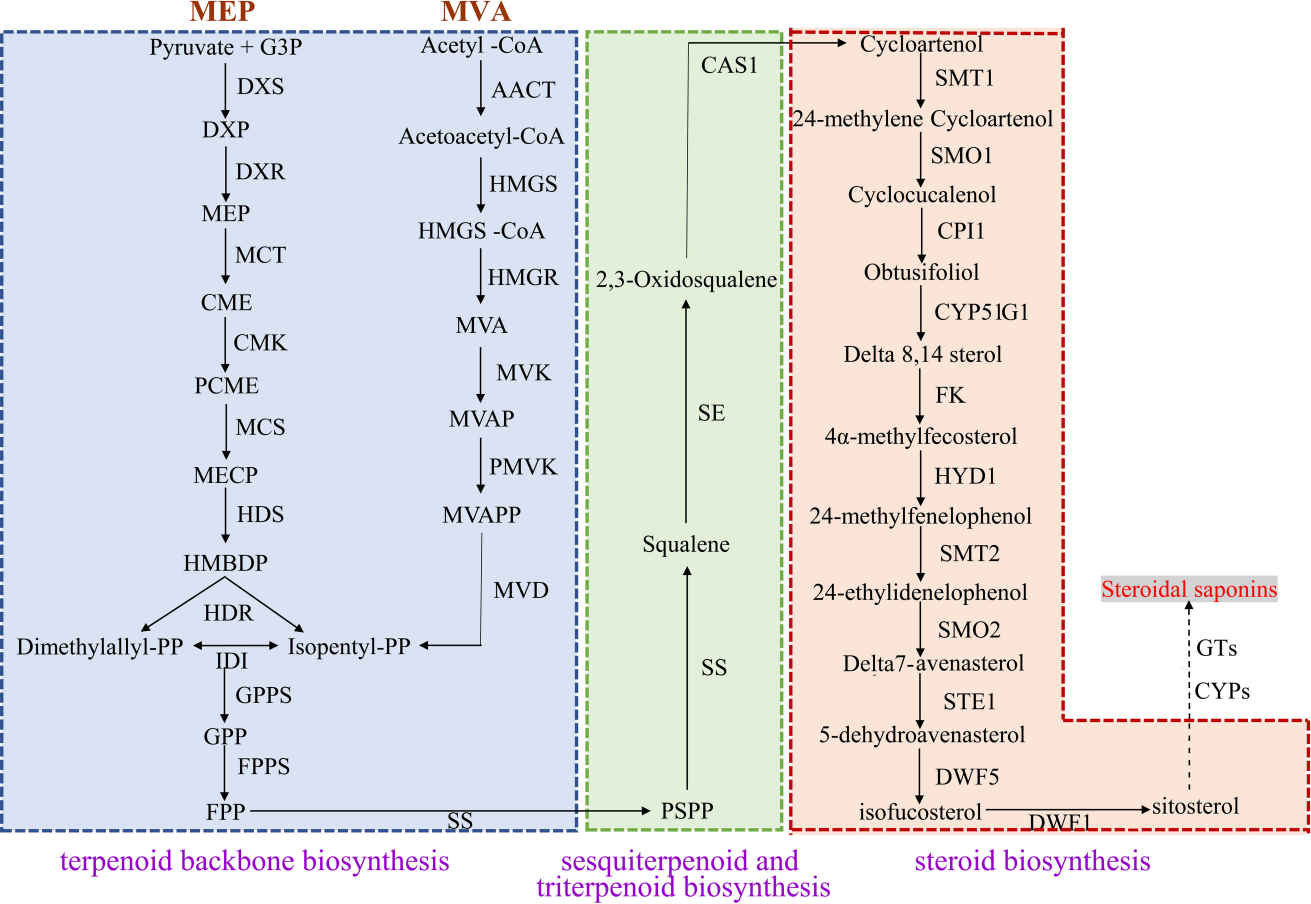
Pathway for steroidal saponins biosynthesis in three *Polygonatum* plants.

**Figure 8:**
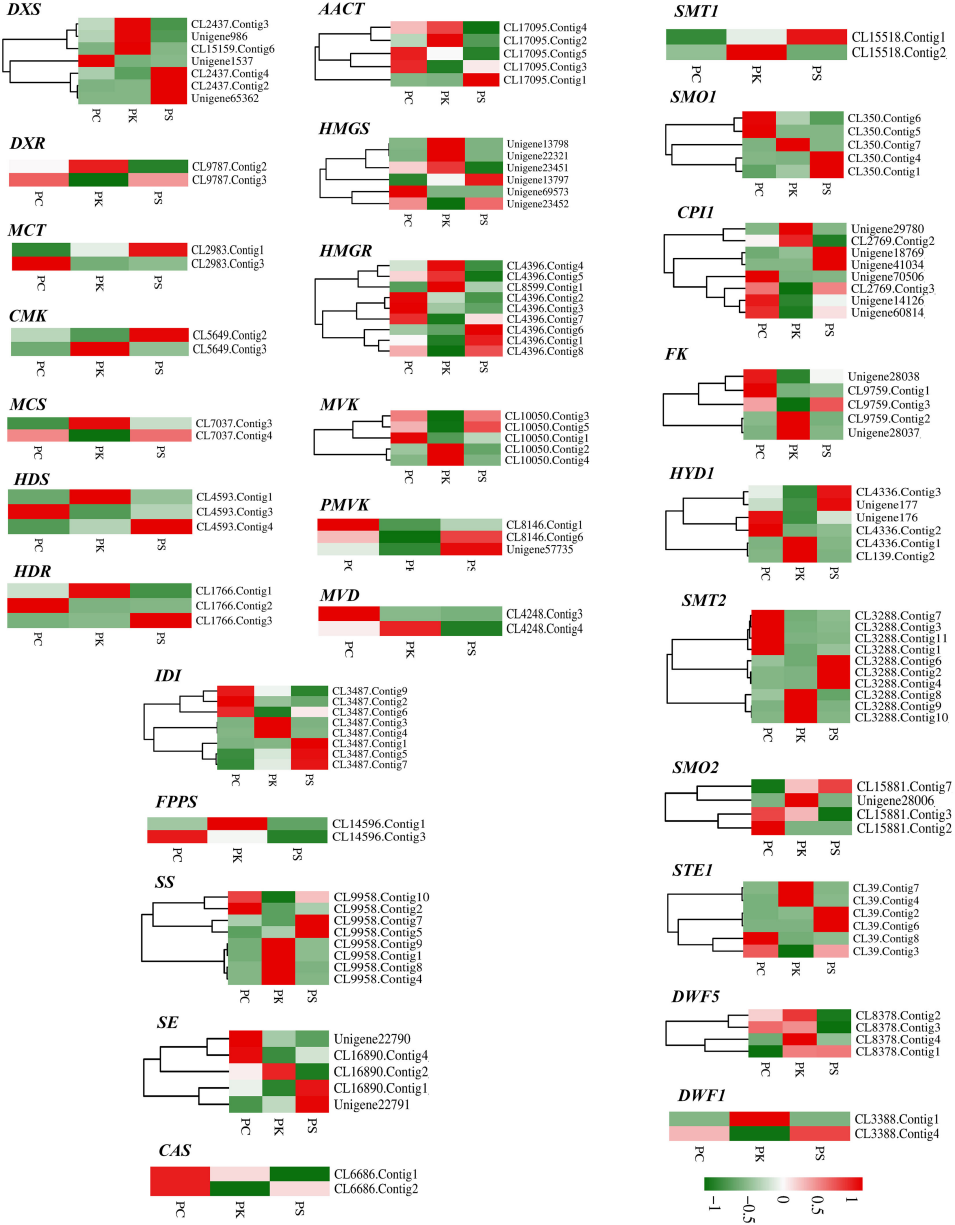
Relative expression patterns of unigenes encoding enzymes involved in steroidal saponins biosynthesis.

In the MVA pathway, 30 DEGs encoding six key enzymes including Acetyl-CoA C-acetyltransferase (AACT, 5 unigenes), Hydroxymethylglutaryl CoA synthase (HMGS, 6 unigenes), HMGR (9 unigenes), Mevalonate kinase (MVK, 5 unigenes), Phosphomevalonate kinase (PMVK, 3 unigenes) and Diphosphomevalonate decarboxylase (MVD, 2 unigenes) were identified. In the MEP pathway, 21 DEGs encoding seven key enzymes were identified, namely 1-deoxy-D-xylulose-5-phosphate synthase (DXS, 7 unigenes), 1-deoxy-D-xylulose-5-phosphate reductoisomerase (DXR, 2unigenes), 2-C-methyl-d-erythritol 4-phosphate cytidylyltransferase (MCT, 2 unigenes), 4-diphosphocytidyl-2-C-methyl-D-erythritol kinase (CMK, 2 unigenes), 2-C-methyl-D-erythritol 2,4-cyclodiphosphate synthase (MCS, 2 unigenes), (E)-4-hydroxy-3-methylbut-2-enyl-diphosphate synthase (HDS, 3 unigenes) and 4-hydroxy-3-methylbut-2-enyl diphosphate reductase (HDR, 3 unigenes). IPP and DMAPP can be converted into each other under the catalysis of Isopentenyl-diphosphate Delta-isomerase (IDI), and then they generate the precursor substance FPP of squalene under the action of GPPS and FPPS. We identified eight DEGs encoding IDI and two DEGs encoding FPPS. In the sesquiterpenoid and triterpenoid biosynthesis and steroid biosynthesis pathways, we obtained 67 DEGs, which encoded 13 key enzymes including SS (8 unigenes), SE (5 unigenes), Sterol 24-C-methyltransferase (SMT1, 2 unigenes), Cycloeucalenol cycloisomerase (CPI1, 8 unigenes), Delta 14-sterol reductase (FK, 5 unigenes), Cholestenol Delta-isomerase (HDY1, 6 unigenes), etc. Notably, two *CAS* genes that catalyzed the formation of Cycloartenol from 2,3-Oxidosqualene were highly expressed in *P. cyrtonema*, while genes encoding SMT1 and DWF1 were expressed at low levels in *P. cyrtonema*. In addition, we found that the gene encoding DWF5 was highly expressed in *P. kingianum* (**Figures 7**, **8**).

### Identification of TFs involved in the biosynthesis of polysaccharides and other secondary metabolites

**Figure 9:**
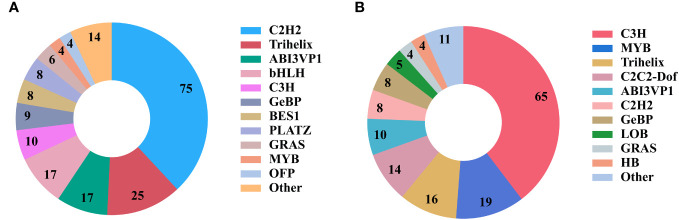
Transcription factors (TFs) involved in metabolic pathways. **(A)** TF families involved in carbohydrate metabolism. **(B)** TF families involved in the metabolism of other secondary metabolites.

TFs can regulate the expression of genes in metabolic pathways and are important regulatory factors for plant development, stress response, and other activities (Riaño-Pachón et al., 2007). A total of 4454 TFs belonging to 57 TF families were identified in *P. sibiricum*, *P. cyrtonema*, and *P. kingianum*. With *P. cyrtonema* as the control group, 767 up-regulated TFs and 847 down-regulated TFs were identified in *P. sibiricum*, and 1010 up-regulated TFs and 1043 down-regulated TFs were identified in *P. kingianum*. In this study, 510 TFs were annotated to MYB TF family, accounting for the largest proportion, followed by AP2-EREBP, C3H, bHLH, and WRKY TF families (**Table 4**). Meanwhile, annotation results showed that 197 TFs from 20 TF families were involved in the carbohydrate metabolism pathway, and 164 TFs from 16 TF families were involved in the biosynthesis of other secondary metabolites (**Figure 9**). By KEGG enrichment analysis, there were 3, 85, 32 and 38 unigenes encoding TFs assigned to fructose and mannose metabolism (ko00051) pathway, galactose metabolism (ko00052) pathway, starch and sucrose metabolism (ko00500) pathway, amino sugar and nucleotide sugar metabolism (ko00520) pathway related to polysaccharide biosynthesis, respectively.

**Table 4:** Classification and number of TF families identified in the DEG database.

TF Family	Number of genes	Number of upregulated genes		Number of downregulated genes	
		PS vs. PC	PK vs. PC	PS vs. PC	PK vs. PC
MYB	510	86	110	94	126
AP2-EREBP	317	54	73	53	62
C3H	309	60	87	66	77
bHLH	276	48	67	58	59
WRKY	234	46	38	35	42
NAC	220	36	51	42	52
FAR1	211	30	43	33	47
G2-like	162	16	33	23	32
C2H2	137	32	32	27	36
Trihelix	130	25	35	25	32
ABI3VP1	128	16	23	19	28
GRAS	113	29	28	24	31
mTERF	111	16	16	22	25
ARF	107	20	37	27	28
SBP	101	18	31	24	32
HSF	87	17	13	22	22
FHA	83	10	16	17	25
C2C2-Dof	72	9	20	13	14
TUB	70	8	20	12	14
Other	1076	191	237	211	259
Total number	4454	767	1010	847	1043

### Bioinformatics analysis of UDP-apiose/xylose synthase genes

AXS catalyzes the ring contraction and closure of UDP-D-glucuronic acid (UDP-GlcA) to produce UDP-Aal and UDP-Xyl. D-apiose (Api) is essential for the development of plant cell walls (Zhao et al., 2020b), and D-xyl is a component of *P. cyrtonema*, *P. sibiricum*, and *P. kingianum* polysaccharides (Zhao et al., 2020a). We identified three DEGs encoding AXS in the transcriptome data.

**Figure 10:**
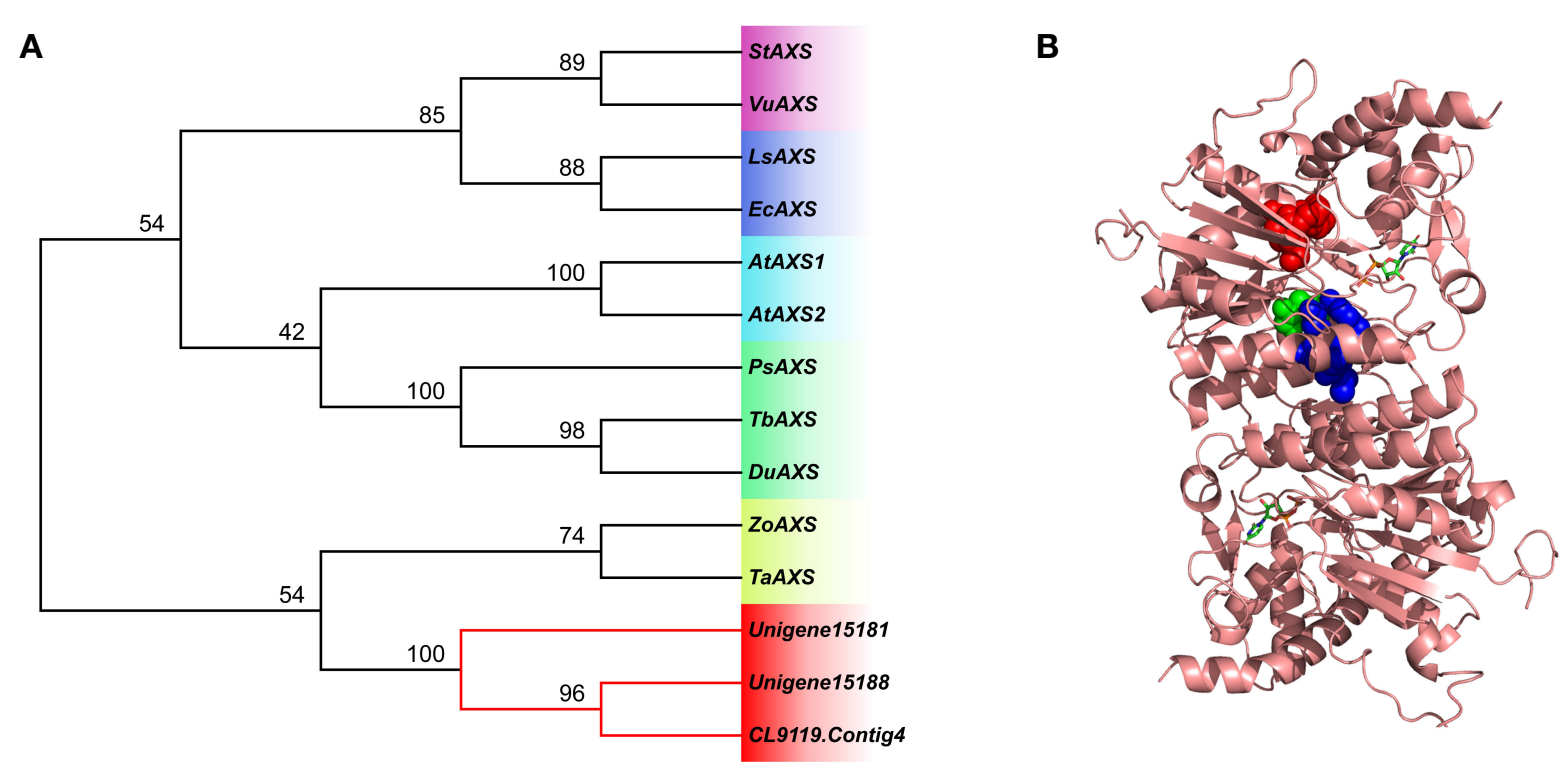
**(A)** Phylogenetic tree showing the three unigenes of AXS. The neighbor-joining phylogenetic trees were constructed using the bootstrap method in MEGA 7.0, and the number of bootstrap replications was 1000. **(B)** Spatial structure models and domain analysis of *AXS* (Unigene15181) gene. Conserved regions, GxxGxxG, ST, and YxxxK, are separately depicted as spheres in red, green, and blue.

Multiple sequence alignment showed that each of the three *AXS* genes had a GxxGxxG motif at the N-terminal, which was thought to be related to cofactor NAD^+^ binding. In addition, sequences contained conserved ST motifs and YxxxK motifs, and YxxxK motifs may play catalytic roles (Thoden et al., 1997). *AXS* (Unigene15181) gene was further selected to create a 3D construction model (**Figures 10**, **S5**; **Table S3**).

### Validation and expression analysis of genes encoding key enzymes

**Figure 11:**
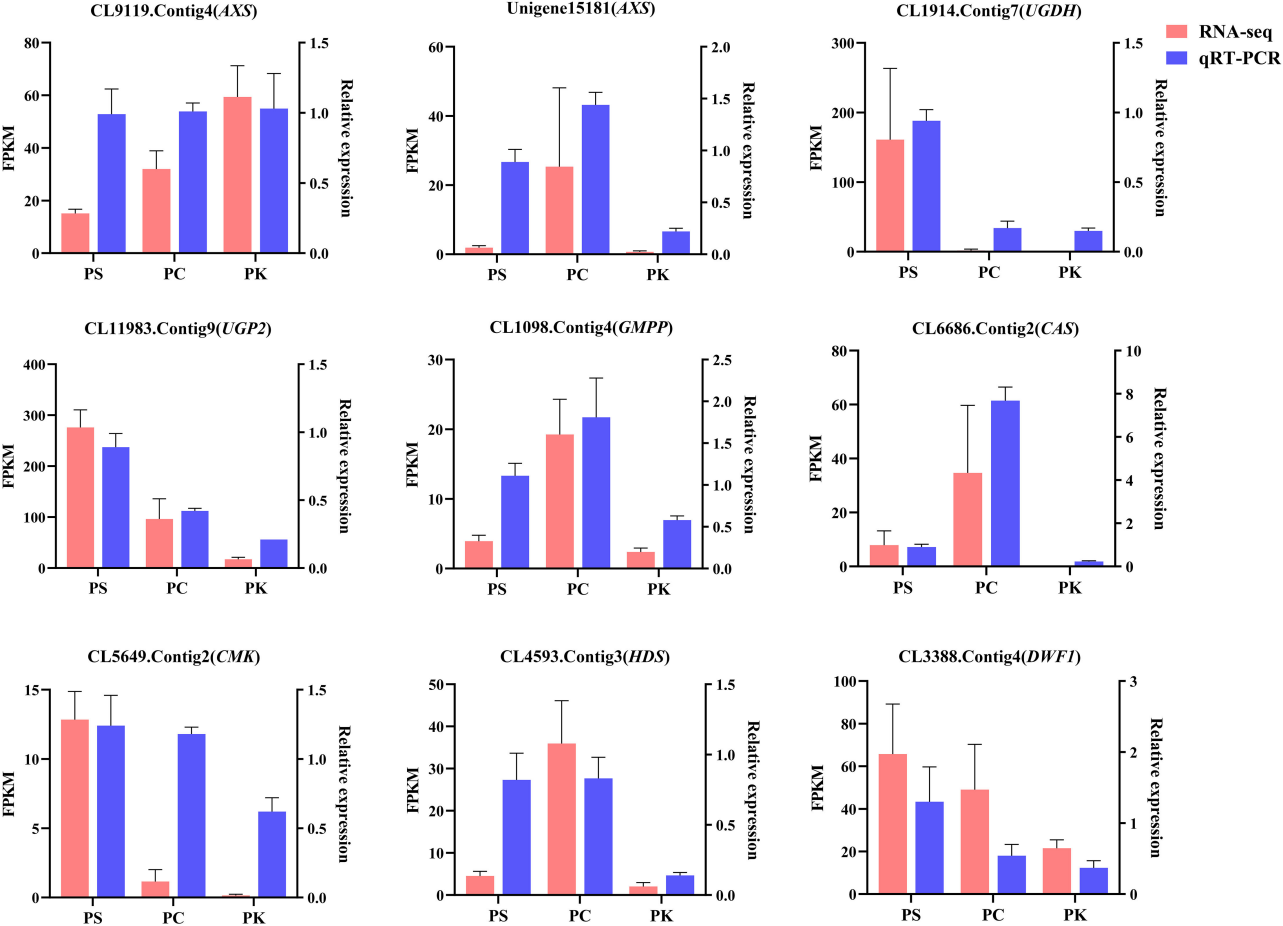
qRT-PCR validation of selected genes. The red bars represent the FPKM values of genes from RNA-seq, and blue bars represent the relative expression determined by qRT-PCR. Data represent mean ± standard error of three replicates. The left Y-axis denotes the FPKM values, and the right Y-axis denotes relative expression levels.

Using *EF-1α2* as the reference gene, 9 DEGs involved in polysaccharide and steroidal saponins synthesis were verified by qRT-PCR, all primers used were listed in **Table S4**. The results showed that the trend of RNA-seq and qRT-PCR data was consistent, indicating that the data of RNA-seq was reliable (**Figure 11**).

## Discussion

The traditional Chinese medicine Huangjing is based on the rhizome of *P. sibiricum*, *P. cyrtonema*, and *P. kingianum*. It is listed as the top grade in the *Ming Yi Bie Lu*, and can be used as food materials. It is a good tonic medicine for both medicine and food. At present, there are no reports on the genome of *P. sibiricum*, *P. cyrtonema*, and *P. kingianum* at home and abroad. High-throughput transcriptome sequencing technology has become an effective means to study the metabolic pathways of active components of polygonati rhizoma and to mine key enzyme genes due to its advantages of convenience and efficiency. The biosynthetic pathways of polysaccharides and steroidal saponins of polygonati rhizoma have been gradually analyzed, but the differences in the expression of key genes in the metabolic pathways of polysaccharides and steroidal saponins of these three plants have not been reported. Therefore, the transcriptome data of these three plants were analyzed in this paper to study the similarities and differences in their biosynthetic pathways, laying a foundation for improving the content of effective components by molecular means.

β-fructofuranosidase belongs to the 32 family of glycoside hydrolase J clan and is the first key enzyme in the synthesis of *polygonatum* polysaccharide (Lammens et al., 2009; Naumoff, 2011). Scholars based on transcriptome data analysis, combined with the polysaccharide content determination results, found that the expression pattern of β-fructofuranosidase and polysaccharide content is positively correlated, in the genus *Polygonatum* another plant *Polygonatum odoratum* also found that most unigenes expression pattern of encoding β-fructofuranosidase is consistent with the content changes (Wang et al., 2017; Zhang et al., 2020; Feng et al., 2022). After antisense inhibition of GMPP, the content of mannose in transgenic potato decreased by 30-50% compared with wild type (Keller et al., 1999). The mannose content of *DoGMP1* isolated from *Dendrobium officinale* was significantly increased after overexpression, and transgenic plants showed better growth under salt stress, suggesting that *GMPP* gene may have potential as a candidate gene to improve plant abiotic stress tolerance (He et al., 2017a). In addition to catalyzing the interconversion of UDP-galactose and UDP-glucose, GalE can also catalyze the interconversion of D-galactose and D-glucose and other free monosaccharides, providing a new scheme for the preparation of rare free monosaccharides (Kim et al., 2011). The analysis of transcriptome data indicates that SacA, GMPP, GALE, and others play important roles in the polysaccharide synthesis pathway of *Polygonatum* plants (Wang et al., 2017; Li et al., 2022). We found that 13 genes encoding SacA, scrK, GMPP, MPI, GMDS, GPI, GALE, and AXS were highly expressed in *P. cyrtonema*, while their expression levels were low in *P. kingianum*, suggesting that they might be putative genes affecting polysaccharide synthesis in Polygonati Rhizoma. *DoPMM* overexpression resulted in 77%, 22%, and 39% increases in polysaccharide content of *Arabidopsis thaliana* transgenic lines #1, #2, and #5 compared to wild type (WT) levels (He et al., 2017b). *Ganoderma* and *Pseudoalteromonas agarivorans* Hao also confirmed that PMM expression level was positively correlated with polysaccharide content (Ju et al., 2022; Zhao et al., 2022). However, the results of this study showed that the expression level of PMM was the highest in *P. kingianum*.

AXS and UDP-xylose synthase (UXS) has the function of converting UDP-D-GlcA to UDP-D-xyl, and AXS can also catalyze UDP-D-GlcA to UDP-D-Api through decarboxylation and rearrangement of the carbon skeleton. The functional expression of *Arabidopsis* AtAXS1 in *Escherichia coli* confirmed that it is a bifunction enzyme (Mølhøj et al., 2003). VIGS of a *Nicotiana benthamiana* homolog of the *AXS* genes resulted in cell death in leaves, obstructed synthesis of RG-II, and abnormal cell wall (Ahn et al., 2006). Many studies have provided evidence that AXS is a key regulator of polysaccharide production. The polysaccharide content of rice root cell walls increased under vanadium stress, and the polysaccharide level of tolerant varieties was higher than that of sensitive varieties. Transcriptome analysis showed that genes encoding AXS were also highly expressed in tolerant varieties (Yuan et al., 2022). The polysaccharide content of *P. cyrtonema* at different growth years was positively correlated with the *AXS* gene expression pattern (Li et al., 2022). We identified three differentially expressed *AXS* genes, and multiple sequence alignments showed that they had conserved motifs GxxGxxG, ST, and YxxxK. The expression pattern of one *AXS* gene (Unigene15181) was consistent with that of polysaccharide content, and its expression level was verified by qRT-PCR.

Some enzymes related to the synthesis of steroidal saponins have been identified, but exploring the enzymes in the pathway of steroidal saponins biosynthesis and the signaling molecules involved in regulation are still the key research areas that need attention (Upadhyay et al., 2018). In this study, 155 genes encoding key enzymes during steroidal saponins synthesis were identified, and 128 DEGs were screened out. Among them, 30 DEGs were associated with MVA pathway, and 21 DEGs were associated with MEP pathway. MVA and MEP pathways may be all involved in the synthesis of saponins (Sun et al., 2017; Upadhyay et al., 2018). The transcriptome analysis of *P. kingianum*, *Asparagus racemosus*, and *Trillium govanianum* showed that monocotyledons seem to produce steroidal saponins through the cycloatenol pathway (Upadhyay et al., 2014; Singh et al., 2017b; Yang et al., 2019). CAS with lupeol synthase (LS), lanosterol synthase (LAS), α-amyrin synthase (AAS), β -amyrin synthase (BAS) belongs to the oxidosqualene cyclases (OCSs) gene family, it can catalyze 2,3-oxidosqualene to produce cycloartenol, the precursor of steroid saponins, sterols, and steroid alkaloids, through chair-boat-chair conformation (Chen et al., 2022). The important functions of *CAS* gene have been identified in a variety of plants. Overexpression of FPPS and RNA interference of CAS in *Panax notoginseng* cells finally led to increased total triterpene saponins content and decreased phytosterols content, and inhibition of CAS expression in Tobacco BY-2 cell suspensions also caused a significant decrease in phytosterol accumulation. We obtained two *CAS* genes in the transcriptome data, which were highly expressed in *P. cyrtonema*, consistent with the high content of saponin than *P. sibiricum* and *P. kingianum*. 

TFs can be combined with specific sequences of DNA, inhibit or enhance the expression of target genes, a TF can regulate the expression of multiple genes, in improving the active ingredient content has more advantages than adjusting the single enzyme (Yuan and Grotewold, 2015). In our study, a total of 4454 TFs belonging to 57 TF families were found, among which the MYB family contained the most TFs, followed by AP2-EREBP, C3H, bHLH, WRKY, etc. MYB is one of the largest TF families in eukaryotes and plays an important role in many physiological processes such as secondary metabolism regulation and stress response in plants. It has been reported that overexpression of the candidate gene *DOMYB75* in *Arabidopsis thaliana* can increase the water-soluble polysaccharide content of seeds by about 14% (He et al., 2019). *AtMYB46* can directly bind to the CSLA9 promoter sequence, and its overexpression can significantly increase mannose content (Kim et al., 2014). A total of 510 MYB TFs were identified, 94 and 126 TFs were up-regulated in *P. cyrtonema* compared with *P. sibiricum* and *P. kingianum*, respectively. These up-regulated genes may be related to the content of polysaccharides and saponins in Polygonati Rhizoma. Yeast one-hybrid assay and electrophoretic mobility shift assay showed that SMBHLH2 and SMWRKY27 might play important roles in sapindite triterpenoid saponin accumulation by directly regulating the transcription of *SMCYP71D-3* (Xu et al., 2022). *Panax notoginseng* PnbHLH1 TF positively regulates the synthesis of triterpenoid saponins (Zhang et al., 2017). Previous studies have shown that TF family members such as MYB (Li and Lu, 2014; Hao et al., 2020), WRKY (Ma et al., 2009; Singh et al., 2017a) and bHLH (Mertens et al., 2016; Jiang et al., 2021) are involved in the regulation of various secondary metabolites in plants; therefore, the TFs identified in this study that were upregulated in *P. cyrtonema* need to be further investigated.

## Conclusion

Comparative transcriptome analysis was conducted using the RNA-seq method on *P. sibiricum*, *P. cyrtonema*, and *P. kingianum*. The key enzymes involved in the biosynthesis pathway of polysaccharide and steroidal saponins of Polygonati Rhizoma were discovered and the DEGs were further analyzed. In this paper, we reported the transcriptome sequencing data of three medicinal plants of the genus *Polygonatum*, and verified the accuracy of the sequencing results by qRT-PCR. The acquired transcriptome data are helpful for the molecular study of polysaccharide and steroidal saponins synthesis pathways in *Polygonatum* SPP.

## Data availability statement

The datasets presented in this study can be found in online repositories. The names of the repository/repositories and accession number(s) can be found below: https://www.ncbi.nlm.nih.gov/, PRJNA974718.

## Author contributions

JML: Writing – original draft. JY: Writing – original draft. JP: Writing – original draft. DW: Writing – original draft. JLL: Writing – original draft. YZ: Writing – review & editing. LZ: Writing – review & editing.
