# Supplementary material for: Transcriptome analysis of three medicinal plants of the genus Polygonatum: identification of genes involved in polysaccharide and steroidal saponins biosynthesis
Source: Front Plant Sci. 2023 Nov 17;14:1293411. doi: 10.3389/fpls.2023.1293411 (PMC10691381; doi:10.3389/fpls.2023.1293411)
Supplement: Supplementary file 1 [file DataSheet_1.docx]

**­­ Table S1** Summary of reads from the RNA-seq data and matches with transcripts.

| Sample | Total Raw Reads (M) | Total Clean Reads (M) | Total Clean Bases(Gb) | Clean Reads Q20(%) | Clean Reads Q30(%) | Clean Reads Ratio(%) |
| --- | --- | --- | --- | --- | --- | --- |
| PC_1 | 45.57 | 42.74 | 6.41 | 97.26 | 92.87 | 93.78 |
| PC_2 | 45.57 | 42.38 | 6.36 | 97.23 | 92.83 | 92.98 |
| PC_3 | 45.57 | 42.6 | 6.39 | 97.16 | 92.63 | 93.47 |
| PK_1 | 45.57 | 43.02 | 6.45 | 97.11 | 92.52 | 94.39 |
| PK_2 | 45.57 | 42.39 | 6.36 | 97.28 | 92.91 | 93.02 |
| PK_3 | 45.57 | 42.7 | 6.41 | 97.18 | 92.68 | 93.7 |
| PS_1 | 47.33 | 43.07 | 6.46 | 97.73 | 93.54 | 91.01 |
| PS_2 | 45.57 | 42.14 | 6.32 | 97.16 | 92.64 | 92.47 |
| PS_3 | 45.57 | 42.86 | 6.43 | 97.22 | 92.8 | 94.05 |

**Table S2** The KEGG pathway annotation.

| Pathway ID | Pathway Name | Gene Number |
| --- | --- | --- |
| ko00010 | Glycolysis / Gluconeogenesis | 1232 |
| ko00020 | Citrate cycle (TCA cycle) | 569 |
| ko00030 | Pentose phosphate pathway | 574 |
| ko00040 | Pentose and glucuronate interconversions | 2179 |
| ko00051 | Fructose and mannose metabolism | 569 |
| ko00052 | Galactose metabolism | 964 |
| ko00053 | Ascorbate and aldarate metabolism | 700 |
| ko00061 | Fatty acid biosynthesis | 402 |
| ko00062 | Fatty acid elongation | 254 |
| ko00071 | Fatty acid degradation | 393 |
| ko00073 | Cutin, suberine and wax biosynthesis | 138 |
| ko00100 | Steroid biosynthesis | 257 |
| ko00130 | Ubiquinone and other terpenoid-quinone biosynthesis | 410 |
| ko00190 | Oxidative phosphorylation | 1080 |
| ko00195 | Photosynthesis | 271 |
| ko00196 | Photosynthesis - antenna proteins | 74 |
| ko00220 | Arginine biosynthesis | 233 |
| ko00230 | Purine metabolism | 825 |
| ko00232 | Caffeine metabolism | 14 |
| ko00240 | Pyrimidine metabolism | 629 |
| ko00250 | Alanine, aspartate and glutamate metabolism | 393 |
| ko00254 | Aflatoxin biosynthesis | 37 |
| ko00260 | Glycine, serine and threonine metabolism | 577 |
| ko00261 | Monobactam biosynthesis | 95 |
| ko00270 | Cysteine and methionine metabolism | 950 |
| ko00280 | Valine, leucine and isoleucine degradation | 554 |
| ko00290 | Valine, leucine and isoleucine biosynthesis | 99 |
| ko00300 | Lysine biosynthesis | 132 |
| ko00310 | Lysine degradation | 390 |
| ko00330 | Arginine and proline metabolism | 522 |
| ko00340 | Histidine metabolism | 221 |
| ko00350 | Tyrosine metabolism | 287 |
| ko00360 | Phenylalanine metabolism | 247 |
| ko00380 | Tryptophan metabolism | 454 |
| ko00400 | Phenylalanine, tyrosine and tryptophan biosynthesis | 467 |
| ko00402 | Benzoxazinoid biosynthesis | 32 |
| ko00410 | beta-Alanine metabolism | 336 |
| ko00430 | Taurine and hypotaurine metabolism | 24 |
| ko00440 | Phosphonate and phosphinate metabolism | 96 |
| ko00450 | Selenocompound metabolism | 214 |
| ko00460 | Cyanoamino acid metabolism | 424 |
| ko00470 | D-Amino acid metabolism | 48 |
| ko00480 | Glutathione metabolism | 608 |
| ko00500 | Starch and sucrose metabolism | 2024 |
| ko00510 | N-Glycan biosynthesis | 605 |
| ko00511 | Other glycan degradation | 466 |
| ko00513 | Various types of N-glycan biosynthesis | 517 |
| ko00514 | Other types of O-glycan biosynthesis | 165 |
| ko00515 | Mannose type O-glycan biosynthesis | 8 |
| ko00520 | Amino sugar and nucleotide sugar metabolism | 1742 |
| ko00523 | Polyketide sugar unit biosynthesis | 2 |
| ko00524 | Neomycin, kanamycin and gentamicin biosynthesis | 33 |
| ko00531 | Glycosaminoglycan degradation | 360 |
| ko00561 | Glycerolipid metabolism | 764 |
| ko00562 | Inositol phosphate metabolism | 876 |
| ko00563 | Glycosylphosphatidylinositol (GPI)-anchor biosynthesis | 312 |
| ko00564 | Glycerophospholipid metabolism | 1064 |
| ko00565 | Ether lipid metabolism | 245 |
| ko00590 | Arachidonic acid metabolism | 173 |
| ko00591 | Linoleic acid metabolism | 197 |
| ko00592 | alpha-Linolenic acid metabolism | 380 |
| ko00600 | Sphingolipid metabolism | 518 |
| ko00601 | Glycosphingolipid biosynthesis - lacto and neolacto series | 3 |
| ko00603 | Glycosphingolipid biosynthesis - globo and isoglobo series | 41 |
| ko00604 | Glycosphingolipid biosynthesis - ganglio series | 226 |
| ko00620 | Pyruvate metabolism | 981 |
| ko00630 | Glyoxylate and dicarboxylate metabolism | 710 |
| ko00640 | Propanoate metabolism | 358 |
| ko00650 | Butanoate metabolism | 154 |
| ko00660 | C5-Branched dibasic acid metabolism | 47 |
| ko00670 | One carbon pool by folate | 209 |
| ko00710 | Carbon fixation in photosynthetic organisms | 604 |
| ko00730 | Thiamine metabolism | 189 |
| ko00740 | Riboflavin metabolism | 221 |
| ko00750 | Vitamin B6 metabolism | 119 |
| ko00760 | Nicotinate and nicotinamide metabolism | 265 |
| ko00770 | Pantothenate and CoA biosynthesis | 337 |
| ko00780 | Biotin metabolism | 210 |
| ko00785 | Lipoic acid metabolism | 44 |
| ko00790 | Folate biosynthesis | 314 |
| ko00860 | Porphyrin metabolism | 380 |
| ko00900 | Terpenoid backbone biosynthesis | 426 |
| ko00901 | Indole alkaloid biosynthesis | 35 |
| ko00902 | Monoterpenoid biosynthesis | 47 |
| ko00903 | Limonene and pinene degradation | 66 |
| ko00904 | Diterpenoid biosynthesis | 183 |
| ko00905 | Brassinosteroid biosynthesis | 101 |
| ko00906 | Carotenoid biosynthesis | 301 |
| ko00908 | Zeatin biosynthesis | 150 |
| ko00909 | Sesquiterpenoid and triterpenoid biosynthesis | 211 |
| ko00910 | Nitrogen metabolism | 370 |
| ko00920 | Sulfur metabolism | 227 |
| ko00940 | Phenylpropanoid biosynthesis | 2544 |
| ko00941 | Flavonoid biosynthesis | 331 |
| ko00942 | Anthocyanin biosynthesis | 29 |
| ko00943 | Isoflavonoid biosynthesis | 114 |
| ko00944 | Flavone and flavonol biosynthesis | 45 |
| ko00945 | Stilbenoid, diarylheptanoid and gingerol biosynthesis | 234 |
| ko00950 | Isoquinoline alkaloid biosynthesis | 196 |
| ko00960 | Tropane, piperidine and pyridine alkaloid biosynthesis | 182 |
| ko00965 | Betalain biosynthesis | 41 |
| ko00966 | Glucosinolate biosynthesis | 21 |
| ko00970 | Aminoacyl-tRNA biosynthesis | 796 |
| ko00996 | Biosynthesis of various alkaloids | 34 |
| ko00999 | Biosynthesis of various plant secondary metabolites | 522 |
| ko01040 | Biosynthesis of unsaturated fatty acids | 174 |
| ko01200 | Carbon metabolism | 2283 |
| ko01210 | 2-Oxocarboxylic acid metabolism | 436 |
| ko01212 | Fatty acid metabolism | 658 |
| ko01230 | Biosynthesis of amino acids | 2212 |
| ko01240 | Biosynthesis of cofactors | 2463 |
| ko01250 | Biosynthesis of nucleotide sugars | 981 |
| ko02010 | ABC transporters | 588 |
| ko03008 | Ribosome biogenesis in eukaryotes | 1227 |
| ko03010 | Ribosome | 2512 |
| ko03013 | Nucleocytoplasmic transport | 2423 |
| ko03015 | mRNA surveillance pathway | 1957 |
| ko03018 | RNA degradation | 1591 |
| ko03020 | RNA polymerase | 946 |
| ko03022 | Basal transcription factors | 573 |
| ko03030 | DNA replication | 723 |
| ko03040 | Spliceosome | 3499 |
| ko03050 | Proteasome | 527 |
| ko03060 | Protein export | 438 |
| ko03410 | Base excision repair | 523 |
| ko03420 | Nucleotide excision repair | 722 |
| ko03430 | Mismatch repair | 411 |
| ko03440 | Homologous recombination | 790 |
| ko03450 | Non-homologous end-joining | 111 |
| ko04016 | MAPK signaling pathway - plant | 2769 |
| ko04070 | Phosphatidylinositol signaling system | 815 |
| ko04075 | Plant hormone signal transduction | 2767 |
| ko04120 | Ubiquitin mediated proteolysis | 1478 |
| ko04122 | Sulfur relay system | 64 |
| ko04130 | SNARE interactions in vesicular transport | 324 |
| ko04136 | Autophagy - other | 416 |
| ko04141 | Protein processing in endoplasmic reticulum | 2504 |
| ko04144 | Endocytosis | 2299 |
| ko04145 | Phagosome | 828 |
| ko04146 | Peroxisome | 876 |
| ko04626 | Plant-pathogen interaction | 3148 |
| ko04712 | Circadian rhythm - plant | 653 |

**Table S3** Relevant AXS sequences for phylogenetic analysis.

| Gene name | Species | Genbank accession number |  |
| --- | --- | --- | --- |
| StAXS | *Solanum tuberosum* | NP_001275341.1 | |
| VuAXS | *Vigna unguiculata* | QCD88085.1 | |
| LsAXS | *Lactuca sativa* | XP_023753060.1 | |
| EcAXS | *Erigeron canadensis* | XP_043629758.1 | |
| AtAXS1 | *Arabidopsis thaliana* | AEC08054.1 | |
| AtAXS2 | *Arabidopsis thaliana* | XNP_563807.1 | |
| PsAXS | *Polypleurum stylosum* | LAE82578.1 | |
| TbAXS | *Terniopsis brevis* | LAF09688.1 | |
| DuAXS | *Dalzellia ubonensis* | LAD63632.1 | |
| ZoAXS | *Zingiber officinale* | XP_042457403.1 | |
| TaAXS | *Triticum aestivum* | XP_044343444.1 | |

**Table S4** Primers of genes for qRT-PCR analysis.

| Gene ID | Gene name | Primers sequence |
| --- | --- | --- |
| \ | *EF-1α2F* | CCCTTCTTGACGCTCTTGAC |
| \ | *EF-1α2R* | GAGCTTCATGGTGCATCTCA |
| CL9119.Contig4_All | *AXS-F* | GATGGTCCAAGCGAGGGTGT |
|  | *AXS-R* | GCCATTAGCACGAGCAGGAT |
| Unigene15181_All | *AXS-F* | AAGGATCACCCTCTACGCCA |
|  | *AXS-R* | CATAGGACCACCTCTGTTTCTCA |
| CL1098.Contig4_All | *GMPP-F* | GAGCTATGGTGGAATGGGATC |
|  | *GMPP-R* | CAGCAGTTCTTTGGTAACAGGGT |
| CL11983.Contig9_All | *UGP2-F* | AAGATCAAGACGCCCACCG |
|  | *UGP2-R* | GAAGAAGAGGCACATTACACCC |
| CL1914.Contig7_All | *UGDH-F* | ACCGTGTCGTTGCGTCCAT |
|  | *UGDH-R* | TGCGGGTCGTAGATGCTGAT |
| CL6686.Contig2_All | *CAS-F* | TTTGGCTTGCCGTACTTGG |
|  | *CAS-R* | AAGCGGACCCACAAACCTCT |
| CL5649.Contig2_All | *CMK-F* | GAGTCCCACTGGATGATAGCAA |
|  | *CMK-R* | CAAAGCAGTTGCGGCATTA |
| CL4593.Contig3_All | *HDS-F* | ACCACTTCCTAATGCTATCGTCCT |
|  | *HDS-R* | ACAGCTAACCGAGTGCCTTCC |
| CL3388.Contig4_All | *DWF1-F* | GGTGTATCCCATCTGGCTCTG |
|  | *DWF1-R* | GCCTCTGGTGGTGCTCAAAC |
