# Supplementary material for: Transcriptome analysis of three medicinal plants of the genus Polygonatum: identification of genes involved in polysaccharide and steroidal saponins biosynthesis
Source: Front Plant Sci. 2023 Nov 17;14:1293411. doi: 10.3389/fpls.2023.1293411 (PMC10691381; doi:10.3389/fpls.2023.1293411)
Supplement: Supplementary file 2 [file DataSheet_2.docx]

**
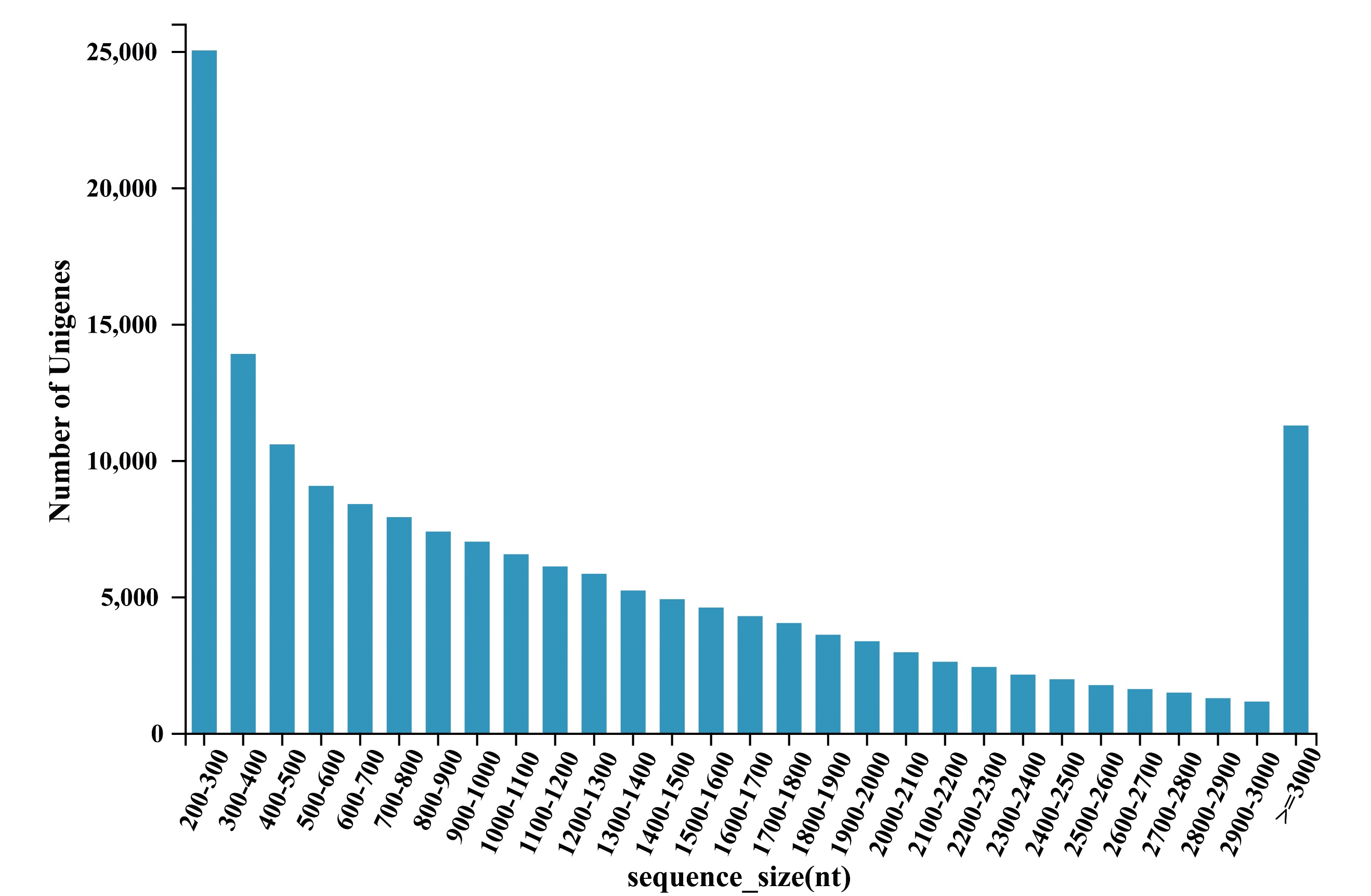
**

**Fig. S1** Sequence length distribution for transcriptome assembly.

­­

**

**

**Fig. S2** KEGG annotation of Polygonati Rhizoma unigenes. **(A)** Pathway classifications for carbohydrate metabolism. **(B)** Pathway classification for the biosynthesis of other secondary metabolites­. **(C)** Pathway classification for metabolism of terpenoids and polyketides


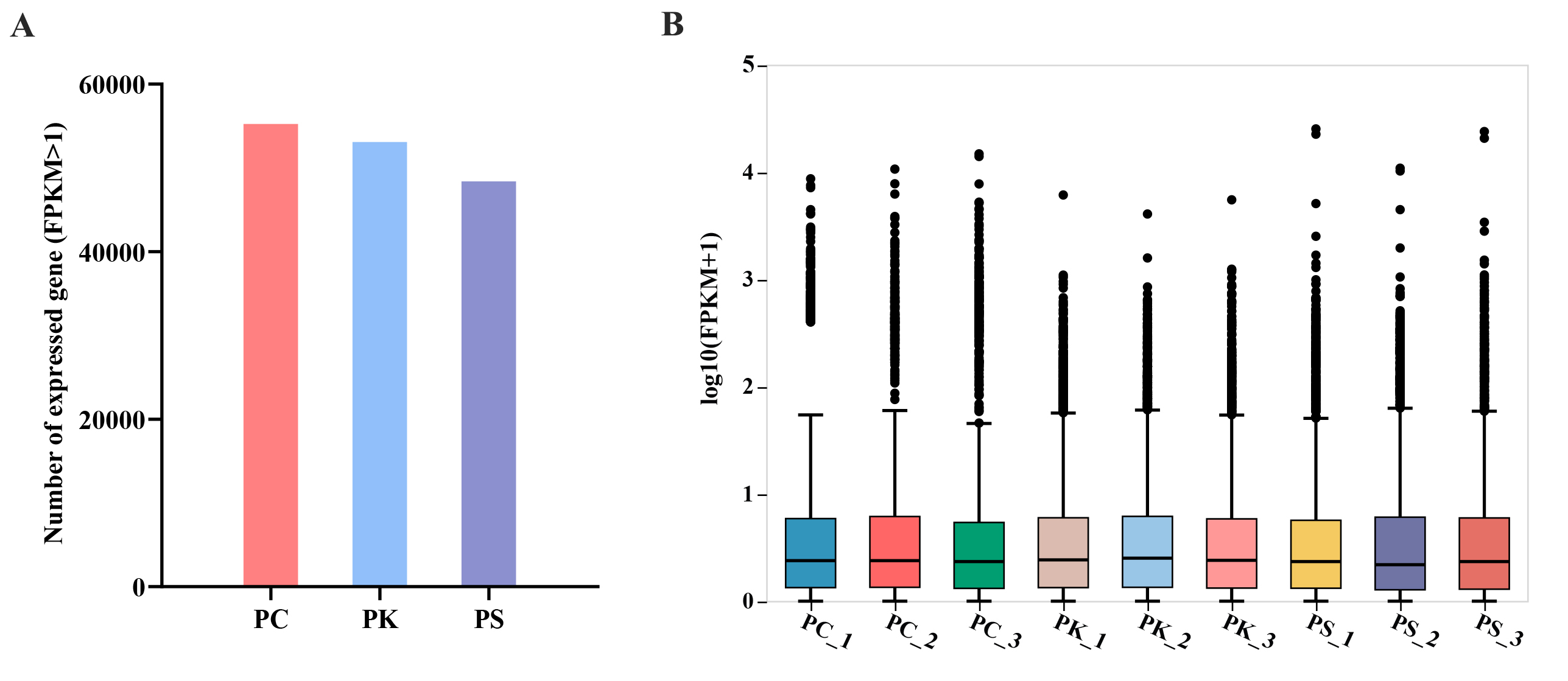


**Fig. S3** Gene expression levels in *P. cyrtonema*, *P. kingianum* and *P. sibiricum*. **(A)** Distributions of expressed unigenes (FPKM > 1) in the three *Polygonatum* species. **(B)** Boxplot of unigenes expressed in the three *Polygonatum* species. X-axis represents the samples, and Y-axis shows the log_10_ (FPKM + 1) values

**

­­**

**Fig. S4** KEGG and GO annotation of DEGs. **(A)** KEGG enrichment analysis of DEGs in *P. cyrtonema* vs. *P. kingianum*. **(B)** GO enrichment analysis of DEGs in *P. cyrtonema* vs. *P. kingianum*. **(C)** KEGG enrichment analysis of DEGs in *P. sibiricum* vs. *P. kingianum*. **(D)** GO enrichment analysis of DEGs in *P. sibiricum* vs. *P. kingianum*. **(E)** KEGG enrichment analysis of DEGs in *P. sibiricum* vs. *P. cyrtonema*. **(F)** GO enrichment analysis of DEGs in *P. sibiricum* vs. *P. cyrtonema*. Asterisks (*) indicates the pathway associated with polysaccharide biosynthesis.

**Fig. S**
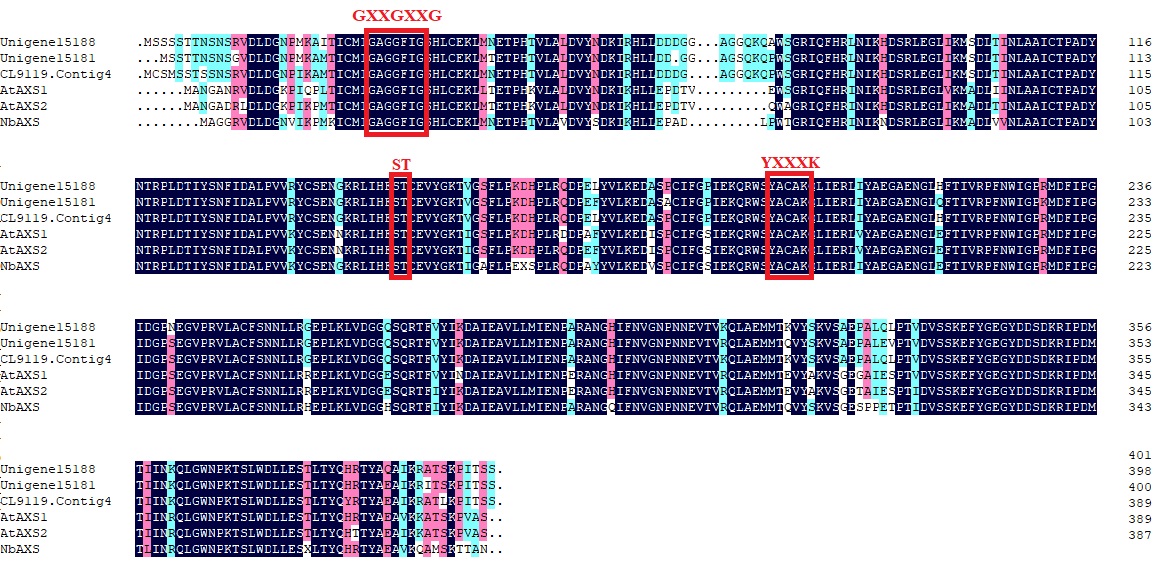
**5** Multiple sequence alignment (dark blue indicates identical amino acids, red and light sky blue indicate similar amino acids, and the identity comparison was performed using DNAMAN software)
